# Legislación sobre seguridad vial en México: un análisis subnacional

**DOI:** 10.26633/RPSP.2017.82

**Published:** 2017-06-19

**Authors:** Ricardo Pérez-Núñez, Delia Ruelas-Valdés, Martha Hijar

**Affiliations:** 1 Secretariado Técnico del Consejo Nacional para la Prevención de Accidentes Secretaría de Salud Ciudad de México México Secretariado Técnico del Consejo Nacional para la Prevención de Accidentes, Secretaría de Salud, Ciudad de México, México.

**Keywords:** Legislación como asunto, prevención de accidentes, accidentes, accidentes de tránsito, México, Legislation as topic, accident prevention, accidents, accidents, traffic, Mexico, Legislação como assunto, prevencão de acidentes, acidentes, acidentes de trânsito, México

## Abstract

**Objetivo.:**

Realizar um diagnóstico da regulamentação federal e estadual para determinar em que medida são cumpridas as recomendações de segurança viária com relação a diferentes fatores de risco e de proteção.

**Métodos.:**

Foi conduzida uma análise descritiva das disposições jurídicas federais e das 32 entidades federativas do México em segurança viária. Foram identificadas as entidades que dispunham de regulamentação legal sobre os principais fatores de risco (excesso de velocidade, consumo de álcool antes de dirigir, uso de celular ao dirigir) e fatores de proteção para acidentes de trânsito (uso de capacete, cinto de segurança e dispositivos de retenção infantil) e analisada sua adequação segundo as recomendações da Organização Mundial da Saúde/Organização Pan-Americana da Saúde (OMS/OPAS). Além disso, são descritos os tipos de sanções aplicadas nestas disposições.

**Resultados.:**

Quase 10% das entidades analisadas dispõem de legislação específica para os seis fatores de risco e de proteção analisados. Observou-se que o fator de risco “consumo de álcool antes de dirigir” e o fator de proteção “uso de capacete” são os mais frequentemente incluídos na legislação estadual. A legislação é adequada em apenas duas entidades federativas (estados): Zacatecas (velocidade de condução) e Jalisco (uso de cinto de segurança, dispositivos de retenção infantil e capacete). A sanção aplicada com maior frequência é a multa.

**Conclusões.:**

É fundamental e prioritário promover uma legislação abrangente contendo disposições adequadas que abordem critérios técnicos e científicos de proteção e prevenção bem como mecanismos apropriados de monitoramento, regulação e cumprimento da legislação.

## Abstract (en)

***Objective*.:**

Study federal and state regulations to determine to what extent road safety recommendations are followed with regard to different risk factors and protective factors.

***Methods*.:**

A descriptive analysis was made of the legal frameworks for road safety at the federal level and in the 32 Mexican states, identifying those that had regulations respecting the main risk factors (speeding, drinking and driving, and cellphone use while driving) and the main protective factors against road injuries (helmet use, seat belt use, and use of child restraint systems). The adequacy of these regulations was analyzed on the basis of Pan American Health Organization/World Health Organization (PAHO/WHO) recommendations. The types of sanctions prescribed in these regulations are also described.

Slightly less than 10% of the entities studied had specific regulations respecting the six protective and risk factors considered. It was noted that “drinking and driving” and “helmet use” are, respectively, the risk factor and protective factor most commonly included in state laws. Adequate regulations were found in only two states: Zacatecas (regarding speeding) and Jalisco (seat belts, child restraint systems, and helmet use). Monetary fines are the most frequently applied sanction.

***Conclusions*.:**

It is a key priority to promote comprehensive laws with provisions that adequately address technical and scientific criteria for protection and prevention, as well as appropriate mechanisms for monitoring, regulation, and sanctions.

## RESUMEN

**Objetivo.:**

*Realizar un diagnóstico de la normativa federal y estatal para determinar en qué medida se siguen las recomendaciones en materia de seguridad vial para distintos factores de riesgo y factores protectores*.

**Métodos.:**

Se realizó un análisis descriptivo de los distintos ordenamientos jurídicos federales y de las 32 entidades federativas de México en el tema de la seguridad vial. Se identificaron aquellas que tenían disposiciones sobre los principales factores de riesgo (exceso de velocidad, alcohol y conducción, uso de celular al conducir) y protectores de lesiones causadas por el tránsito (uso de casco, uso de cinturón de seguridad y uso de sistemas de retención infantil) y se analizó si estas eran adecuadas, con base en las recomendaciones de la Organización Mundial de la Salud/Organización Panamericana de la Salud (OMS/OPS). Además, se describen los tipos de sanciones empleados en dichos ordenamientos.

**Resultados.:**

Poco menos de 10% de las entidades analizadas cuentan con normatividad específica para los seis factores protectores y de riesgo analizados. Se observó que el factor de riesgo “alcohol y conducción” y el factor protector “uso de casco” son los que están más considerados en las leyes estatales. Solo en dos entidades federativas (estados) la normatividad es adecuada: en Zacatecas (en el tema de velocidad) y en Jalisco (cinturón de seguridad, sistemas de retención infantil y uso de casco). La sanción aplicada con mayor frecuencia es la multa económica.

**Conclusiones.:**

Es fundamental y prioritario impulsar leyes integrales que contemplen disposiciones adecuadas que aborden criterios técnicos y científicos de protección y prevención, así como los mecanismos apropiados para vigilar, regular y sancionar.

Distinta evidencia ha documentado los efectos preventivos de la aplicación rigurosa de las legislaciones para diversos factores de riesgo y protectores de lesiones causadas por el tránsito (1–2). Reconociendo este potencial preventivo, la Organización Mundial de la Salud (OMS) promueve desde 2004 la adopción y cumplimiento de leyes en al menos cinco temas —velocidad, alcohol y conducción, cinturones de seguridad, sistemas de retención infantil (SRI), así como cascos para ciclistas y motociclistas— como una buena práctica (1). Para evaluar en qué medida los países miembros habían implementado sus recomendaciones, durante 2008 la OMS realizó una encuesta global sobre el estado de la seguridad vial en el mundo y publicó un informe mundial (3) y otro para la Región de las Américas (4). Esto ha permitido dar seguimiento a los avances mundiales a través de dos ediciones más, una en 2013 (5) y la otra en 2015 (6). Esta información permitió conocer por primera vez el estado de las legislaciones nacionales en materia de seguridad vial a nivel global y evaluar los avances en la Década de Acción por la Seguridad Vial 2011–2020 promovida por Naciones Unidas (7).

El análisis realizado en estos reportes muestra a México con resultados contradictorios. Mientras que en el primero y segundo informes se establecía que sí existía una ley nacional para el uso de casco en motociclistas (reportada como adecuada), en el tercero la respuesta fue negativa. Los dos primeros informes dicen que sí existía una legislación nacional para el uso de cinturón de seguridad, pero el tercero menciona que no; mientras que en el primer informe fue reportada como adecuada, para el segundo se registró como inadecuada. En el primer informe se respondió que sí existía una ley nacional para SRI, en el segundo se dijo que era subnacional y para el tercero la respuesta fue negativa (3, 5, 6, 8).

Esto pudo ser resultado de dos factores. El primero, que la forma de preguntar o de interpretar las preguntas por las personas invitadas a participar en las diferentes rondas, que no fueron las mismas en todas ellas, pudo haber influido en las respuestas. El segundo, la existencia de una normatividad de tránsito en carreteras y puentes de jurisdicción federal (9), que si bien es de cobertura nacional, no aplica para vialidades de jurisdicción estatal y municipal. Esto generó confusión en cuanto a si esta disposición debía ser considerada como “ley nacional” o no.

La Constitución Política de los Estados Unidos Mexicanos establece en su artículo 115, fracción III, inciso H (10) que, si bien los 2 454 municipios que existen (11) tienen a su cargo la regulación del tránsito municipal, sin perjuicio de su competencia constitucional, deberán observar lo dispuesto por las leyes federales y estatales (10). Así, como una forma de homologar los criterios empleados en sus municipios, las autoridades federales y de las 32 entidades federativas pueden definir estándares de seguridad a través de sus leyes y reglamentos, que deben ser retomadas por los municipios.

En materia del tránsito y seguridad vial, no se cuenta con ordenamiento alguno de observancia general para todo el territorio nacional. Sin embargo, existen entidades federativas que han logrado avances notables en materia de regulación del tránsito, de allí que las mediciones realizadas por la OMS en sus informes globales no permiten entender de manera adecuada la realidad de países como México, aunque representan un excelente abordaje metodológico para países con un gobierno central.

En un afán por entender mejor la situación actual de la legislación en seguridad vial en México y buscar cómo contribuir a su mejoramiento, el presente documento ofrece un diagnóstico de la normativa federal y estatal para determinar en qué medida se siguen las recomendaciones en materia de seguridad vial para sus principales factores de riesgo y protectores.

## MATERIAL Y MÉTODOS

### Diseño y población de estudio

Con base en el abordaje propuesto por la OMS (12), se realizó un análisis secundario de los principales ordenamientos jurídicos en materia de seguridad vial (leyes y reglamentos de tránsito o movilidad) vigentes al 1 de enero de 2016 de las 32 entidades federativas de México y del nivel federal. Estos fueron descargados de las páginas web de sus instituciones o solicitados directamente a las autoridades encargadas de regular el tema. En el cuadro 1 se menciona el material analizado para cada entidad.

### Recolección de información

Una persona con formación en Derecho, previamente capacitada en seguridad vial, revisó los ordenamientos jurídicos usando la metodología establecida por OMS (12). Mediante una lista de chequeo, identificó si existía alguna disposición para los factores protectores o de riesgo de interés y recabó distintos atributos tal como estaban literalmente expresados en los ordenamientos jurídicos para luego evaluar si dicha disposición era adecuada (12). Las disposiciones se consideraron adecuadas si seguían las recomendaciones de la Organización Panamericana de la Salud/Organización Mundial de la Salud (OPS/OMS) (4, 12) con base en los siguientes criterios:

#### Exceso de velocidad.

Se evaluó si los límites máximos de velocidad para conducir no excedían los siguientes parámetros:

- Vías rápidas: 110 km/h.
- Vías primarias: 50 km/h.
- Vías secundarias: 30 km/h.
- Zonas escolares: 20 km/h.

Además, se evaluó si la detección de la velocidad se hacía mediante radar o fotoinfracción y si se preveía alguna sanción como multa o penalización en el sistema de puntos. Un criterio más relajado consideró solo el límite máximo en vías primarias, tal como lo hiciera OPS/OMS en los informes mundiales (3–5).

#### Alcohol y conducción.

Se evaluó si se definía a través de una tasa de concentración que no excedera los siguientes límites:

- 0,25 mg/L en aire espirado o 0,05 g/dL en sangre.
- En conductores jóvenes o noveles: 0,1 mg/L en aire espirado o 0,02 g/dL en sangre.

Además, se evaluó si la forma de captación era a través de operativos policiales con uso de alcoholímetros como mecanismo de control, si se sancionaba la negativa a practicarse la alcoholimetría y si a los conductores que excedían el límite establecido se les sancionaba con multa, arresto administrativo o retiro de vehículo.

#### Uso de celular al conducir.

Se evaluó si se establecía este en forma explícita, se especificaba además el texteo^2^ como inapropiado y si se sancionaba su uso con multa o penalización en el sistema de puntos. Un criterio más relajado consideró solo el uso de celular y su sanción correspondiente.

#### Uso de cinturón de seguridad.

Se evaluó si se establecía la obligatoriedad de utilizarlo por todos los pasajeros (conductor, pasajeros de adelante y atrás), si se describía la forma adecuada de su uso y si se sancionaba su no uso con multa o penalización en el sistema de puntos.

#### Uso de sistemas de retención infantil.

Se evaluó si se establecían criterios de estatura (< 135 cm), edad (< 13 años) o peso (< 36 kg) para su uso, si se establecían los tipos de SRI a utilizar, si se establecía la necesidad de que estuvieran certificados y si su no uso se sancionaba con multa o penalización en el sistema de puntos.

#### Uso de casco en motociclistas.

Se evaluó si se establecía la forma adecuada de utilizarlo (colocado y abrochado o ajustado), si se reconocía la importancia de que el casco estuviese certificado, si se establecía la obligatoriedad de su uso por todos los ocupantes de la motocicleta y si su no uso se sancionaba mediante multa o penalización en el sistema de puntos.

### Análisis de la información

Se integró una base de datos para realizar un análisis descriptivo de las legislaciones nacionales en el que se calcularon frecuencias y porcentajes para la existencia y adecuación de ordenamientos para los seis factores de interés. Además, se realizó un análisis comparativo de las sanciones establecidas en los ordenamientos jurídicos analizados.

**CUADRO 1.** Fuentes de información consultadas en cada entidad federativa

Entidad federativa	Ley	Reglamento
Nivel Federal	NA	**Reglamento de Tránsito en caminos y puentes de jurisdicción federal.** Publicado en el Diario Oficial de la Federación el 22 de noviembre de 2012.
Aguascalientes	**Ley de Vialidad del Estado de Aguascalientes.** Última reforma el 31 de diciembre de 2012.	NA
Baja California	La regulación es de carácter municipal	
Baja California Sur	**Ley de Tránsito Terrestre del Estado y Municipios de Baja California Sur.** Publicada en el Boletín Oficial del Gobierno del Estado el 20 de marzo de 2005; última reforma el 30 de noviembre de 2013.	NA
Campeche	Ley de Vialidad, Tránsito y Control Vehicular del Estado de Campeche. Última reforma el 5 de diciembre de 2014.	**Reglamento de la Ley de vialidad, tránsito y control vehicular del Estado de Campeche.** Publicado en el Periódico Oficial del Estado el 9 de enero de 2009.
Chiapas	Ley de Transportes del Estado de Chiapas. Publicada en el Periódico Oficial del Estado el 24 de junio de 1998; última reforma el 30 de mayo de 2014.	**Reglamento de tránsito para el Estado de Chiapas.** Publicado en el Periódico Oficial del Estado el 4 de junio de 2014.
Chihuahua	**Ley de vialidad y tránsito del Estado de Chihuahua.** Última reforma el 7 de febrero de 2015.	**Reglamento de la ley de vialidad y tránsito del Estado de Chihuahua.** Última reforma el 23 de agosto de 2013.
Coahuila	Ley de tránsito y transporte del Estado de Coahuila. Última reforma el 23 de setiembre de 2014.	**Reglamento de la Ley de tránsito y transporte del Estado de Coahuila.** Se desconoce su vigencia.
Colima	Ley de transporte y seguridad vial del Estado de Colima. Última reforma el 20 de agosto de 2009.	**Reglamento de vialidad y transporte.** Publicado en el Periódico Oficial del Estado el 13 de julio de 2002. Se desconoce su vigencia.
Ciudad de México	Ley de movilidad del Distrito Federal. Publicada en la Gaceta Oficial del Distrito Federal el 14 de julio de 2014.	**Reglamento de tránsito del Distrito Federal.** Publicado en la Gaceta Oficial del Distrito Federal el 17 de agosto de 2015.
Durango	Ley de tránsito para los municipios del Estado de Durango. Última reforma el 16 de febrero de 2014.	NA
Estado de México	Código Administrativo del Estado de México	**Reglamento de tránsito del Estado de México**
Guanajuato	Ley de tránsito y transporte del Estado de Guanajuato. Última reforma el 7 de junio de 2013.	**Reglamento de la Ley de tránsito y transporte del Estado de Guanajuato.** Se desconoce su vigencia.
Guerrero	Ley de transporte y vialidad del Estado de Guerrero. Última reforma el 27 de octubre de 2009. Se desconoce su vigencia.	**Reglamento de la Ley de transporte y vialidad del Estado de Guerrero.** Se desconoce su vigencia.
Hidalgo	**Ley de vías de comunicación y tránsito para el Estado de Hidalgo.** Última reforma el 6 de agosto de 2001.	NA
Jalisco	**Ley de movilidad y transporte del estado de Jalisco.** Publicado en el Periódico Oficial del Estado el 10 de agosto de 2013; sin reforma.	**Reglamento de la Ley de movilidad y transporte del estado de Jalisco.** Sin reforma.
Michoacán	Ley de tránsito y vialidad del Estado de Michoacán de Ocampo. Última reforma el 23 de agosto de 2007.	**Reglamento de tránsito y vialidad del Estado de Michoacán de Ocampo.** Última reforma el 14 de marzo de 2007.
Morelos	Ley de tránsito del Estado de Morelos. Última reforma el 22 de abril de 2015.	**Reglamento de la Ley de tránsito del Estado de Morelos.**Última reforma el 27 de marzo de 2013.
Nayarit	**Ley de tránsito y transporte del Estado de Nayarit.** Última reforma el 18 de agosto de 2014.	NA
Nuevo León	**Ley que regula la expedición de licencias para conducir en el Estado de Nuevo León.** Última reforma el 29 de octubre de 2014. **Ley para la prevención y combate al abuso del alcohol y de regulación para su venta y consumo para el Estado de Nuevo León.** Última reforma el 29 de octubre de 2014.	NA
Oaxaca	Ley de tránsito reformada. Última reforma el 9 de julio de 1999.	**Reglamento de la Ley de tránsito reformada.** Publicado en el Periódico Oficial el 31 de marzo de 1973.
Puebla	Ley de vialidad del Estado Libre y Soberano de Puebla. Última reforma el 30 de diciembre de 2013.	**Reglamento de la Ley de vialidad para el Estado Libre y Soberano de Puebla.** Última reforma el 19 de julio de 2013.
Querétaro	Ley de tránsito del Estado de Querétaro. Última reforma 22 de junio de 2012.	**Reglamento de la Ley de tránsito del Estado de Querétaro.**Última reforma 25 de enero de 2002.
Quintana Roo	**Ley de tránsito, transporte y explotación de vías carreteras del Estado de Quintana Roo.** Última reforma el 28 de mayo de 2015.	NA
San Luis Potosí	**Ley de tránsito del Estado de San Luis Potosí.** Última reforma el 16 de mayo de 2015.	NA
Sinaloa	**Ley de tránsito y transporte del Estado de Sinaloa.** Última reforma el 30 de enero de 2015.	NA
Sonora	**Ley de tránsito del Estado de Sonora.** Última reforma el 11 de diciembre de 2014.	NA
Tabasco	**Ley general de tránsito y vialidad del Estado de Tabasco.**Última reforma el 9 de abril de 2015.	**Reglamento de la Ley general de tránsito y vialidad del Estado de Tabasco.** Última reforma el 12 de mayo de 2007.
Tamaulipas	**Ley de tránsito del Estado de Tamaulipas.** Última reforma el 25 de noviembre de 2014.	**Reglamento de tránsito del Estado de Tamaulipas.** Última reforma el 2 de diciembre de 2010.
Tlaxcala	**Ley de Comunicaciones y Transporte.** Última reforma no especificada.	**Reglamento de la Ley de comunicaciones y transporte.** Última reforma no especificada.
Veracruz	**Ley número 561 de Tránsito y Seguridad Vial del Estado de Veracruz de Ignacio de la Llave.** Publicada el 13 de abril de 2015	**Reglamento de la Ley 561 de tránsito y seguridad vial del Estado de Veracruz de Ignacio de la Llave.** Publicado en la Gaceta Oficial del Estado el 16 de junio de 2015.
Yucatán	**Ley de tránsito y vialidad del Estado de Yucatán.** Última reforma el 19 de diciembre de 2011.	**Reglamento de la Ley de Tránsito del Estado de Yucatán.** Sin reforma.
Zacatecas	**Ley de transporte, tránsito y vialidad del Estado de Zacatecas.**Publicada en el Periódico Oficial del Estado el 21 de noviembre de 2013.	**Reglamento de la Ley de transporte, tránsito y vialidad del Estado de Zacatecas.** Publicado en el Periódico Oficial del Estado el 5 de julio de 2014.

***NOTA***: cuadro de elaboración propia. Si bien se revisaron todos los documentos contenidos en este cuadro, en negrita aparecen los ordenamientos que contenían las disposiciones que se tomaron en cuenta para el análisis.

## RESULTADOS

**FIGURA 1.**
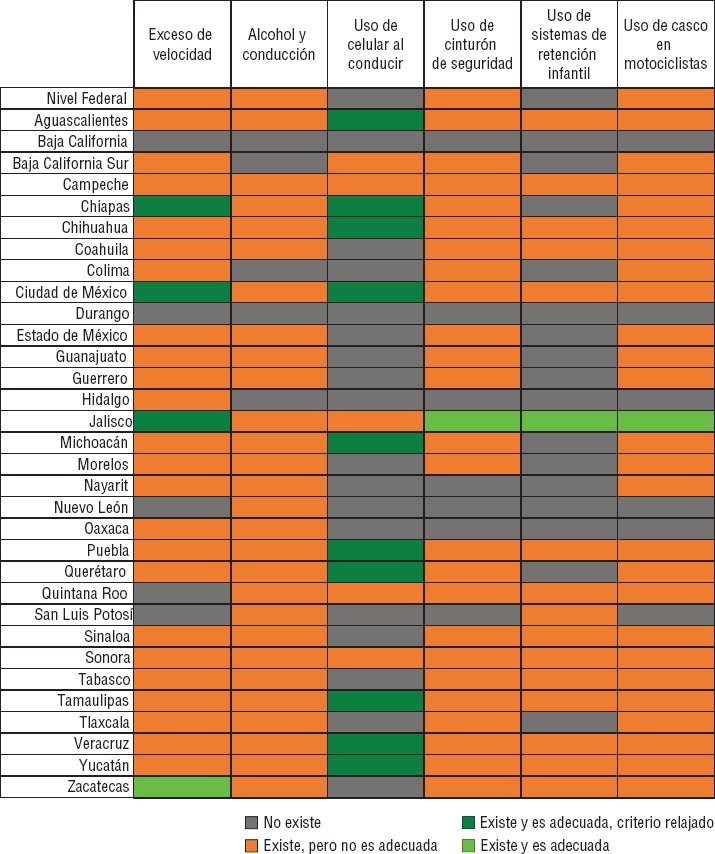
Análisis de la normatividad en seguridad vial y su adherencia a recomendaciones internacionales por factor de riesgo y entidad federativa, México 2016.

**FIGURA 2.**
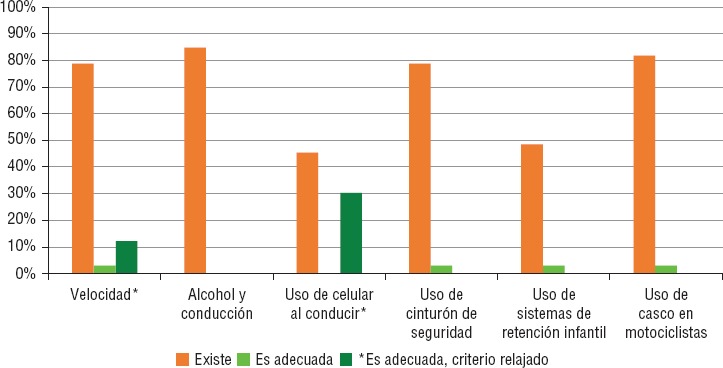
Análisis subnacional de la legislación sobre los principales factores de riesgo y protectores de lesiones causadas por el tránsito en México, 2016.

Poco menos de 10% del total de entidades analizadas tienen normatividad específica para los seis factores analizados; sin embargo, ninguna de ellas tiene todos los factores de forma adecuada (figura 1). En contraparte, las entidades federativas de Baja California y de Durango no cuentan con alguna disposición legal que atienda al menos uno de los seis factores analizados. Hidalgo, Nuevo León y Oaxaca solo abordan uno de ellos. En la figura 2 se muestra el panorama de la legislación en seguridad vial en México.

En la figura 1 se puede apreciar que el exceso de velocidad está considerado en 28 de las 32 entidades federativas del país y el nivel federal. Sin embargo, solo en Zacatecas la legislación es adecuada. Al considerar solo los límites de velocidad en zonas urbanas, se observa que también Chiapas, Ciudad de México y Jalisco cuentan con legislaciones adecuadas.

En el cuadro 2 se mencionan los criterios específicos que no cumple cada entidad. Mientras que algunas fallan en especificar límites de velocidad para vías rápidas, secundarias o zonas escolares, la gran mayoría establece límites mayores a los recomendados. Una omisión común en 21 de las 28 entidades en la que se considera el exceso de velocidad fue la falta de especificación del recurso empleado para su detección (radar o fotoinfracción). Por último, no se establece una sanción adecuada en 18% de las entidades que cuentan con normatividad para exceso de velocidad.

También el factor “alcohol y conducción” fue incluido en 85% de las normativas analizadas. Sin embargo, en ninguna entidad del país fue adecuada. En Morelos y Jalisco no se cumple la especificación de la tasa máxima permitida para conductores jóvenes y noveles, en Yucatán la tasa de concentración de alcohol es mayor a la recomendada (0,8 mg/100 mL en sangre o 0,4 mg/L en aire espirado). Esto impidió que dichas entidades fueran consideradas con adecuada legislación.

***Nota:*** elaboración propia a partir de los resultados presentados.

***Nota:*** figura de elaboración propia a partir de los resultados presentados. El porcentaje corresponde al número de entidades federativas que muestran avance del total de entidades analizadas (32 entidades federativas y el nivel federal). Los criterios para definir si es adecuada incluyendo todos los elementos necesarios o si es adecuada con un criterio relajado se presentan con detalle en la sección de metodología.

El criterio que menos se cumple en el país es el nivel máximo de concentración de alcohol permitido; 25 de las 28 entidades federativas que norman este factor de riesgo establecen niveles por arriba de los recomendados. De igual forma, 24 de las 28 entidades tampoco establecen una tasa inferior para conductores jóvenes o nóveles. En este caso, las excepciones fueron Chihuahua, Estado de México, Yucatán y Zacatecas. Por último, el establecimiento de sanciones para infractores y para quienes se niegan al control policial solo se establece adecuadamente en siete (25%) entidades federativas.

El uso de celular al conducir fue el factor de riesgo menos abordado por los instrumentos legales, con solo 45% de las entidades. El texteo, como distractor, no fue incluido en algún ordenamiento. Sin contar el texteo, en 10 de las 15 entidades que cuentan con esta disposición es adecuada.

El uso de cinturón se incluye en 79% de las legislaciones y reglamentaciones de seguridad vial, aunque solo en Jalisco son adecuadas. La mayor brecha se observa en la definición exacta del adecuado uso de este dispositivo: solo dos entidades lo especificaron. Siete y nueve de las 26 entidades que incluyen este factor protector no especifican que deben utilizarlo todos los pasajeros o no establecen la sanción correspondiente, respectivamente.

El uso de SRI se incluye en las disposiciones de 16 entidades federativas (48%). Jalisco es el único estado en donde se aborda este factor de forma adecuada. El criterio que menos se cumple es el requerimiento de certificación de dichos dispositivos de seguridad. La definición de quiénes lo deben de utilizar solo se incluyó en cinco de las 16 entidades, y el tipo de dispositivo recomendado para distintos grupos solo se estableció en seis. Por último, la sanción no fue adecuada en 50% de los casos.

El uso de casco en motociclistas fue el segundo factor incluido con mayor frecuencia (82%), aunque solo en Jalisco fue adecuado. Se establecen estándares de seguridad del casco que deben utilizar los motociclistas en Tabasco y Veracruz, además de Jalisco. La normatividad en Tabasco hubiera sido considerada adecuada, si se hubiese especificado la forma en cómo se debe utilizar el casco. Esto último no se realizó en 23 de los 27 casos. Cuatro entidades no especifican la necesidad de que tanto pasajeros como conductores lo utilicen y nueve no establecen la sanción correspondiente de manera adecuada.

**FIGURA 3.**
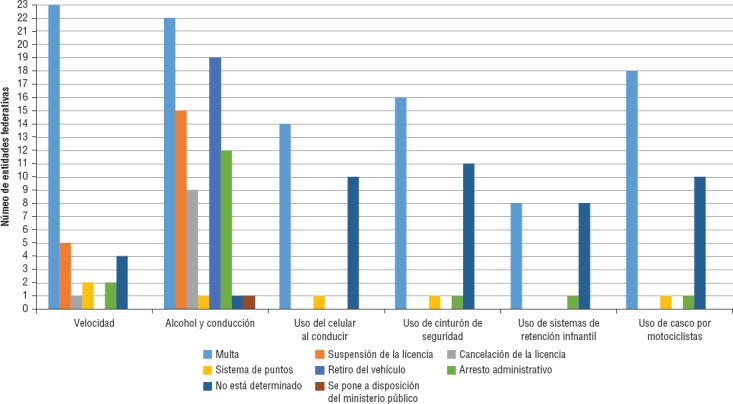
Tipo de sanciones establecidas en los ordenamientos jurídicos en México, 2016.

La multa económica es el mecanismo más utilizado para sancionar a los infractores (figura 3). Las multas se establecieron comúnmente en términos de días de salarios mínimos vigentes (SMV)^3^ o, en la Ciudad de México, en términos de la “unidad de cuenta de la Ciudad de México”. El monto de cada salario o unidad para 2015 son bastante similares y varían entre $68,28 y $70,10^4^ (aproximadamente 4 USD^5^), por lo que nos referiremos a ellos como SMV. Oaxaca es una excepción, pues establece cifras exactas: $100 por uso de celular al conducir, $200 por exceso de velocidad y $400 por conducir bajo el influjo del alcohol.

Hay variaciones importantes por factor de riesgo, nivel de trasgresión (a mayor gravedad, mayor multa) y entidad federativa. La velocidad se sanciona entre 2 y 70 SMV (en Colima y Veracruz, respectivamente) y en Hidalgo se cobra $50 por cada kilómetro excedido. Las multas por conducir bajo los efectos del alcohol pueden ir de 1 SMV en Chiapas a 600 SMV en Nuevo León; por utilizar el celular de 1 SMV en Chiapas a 45 SMV en Veracruz; por no usar cinturón de 1 SMV en Chiapas a 50 SMV en Veracruz; por no utilizar sistema de retención infantil de 4 SMV en Chihuahua a 50 SMV en Veracruz y, finalmente, por no utilizar el casco en motociclistas de 1 SMV en Chiapas a 60 SMV en carreteras federales.

La suspensión de la licencia es una sanción que suele estar asociada a la reincidencia de velocidad excesiva (en Morelos, Nayarit, Sonora y Tamaulipas) o por alcohol y conducción (en Campeche, Chiapas, Coahuila, Estado de México, Guerrero, Jalisco, Morelos, Nayarit, Nuevo León, Oaxaca, Querétaro, Tabasco y Tamaulipas). En Tabasco, una colisión por exceso de velocidad supone la suspensión de la licencia. La cancelación definitiva de licencias para conducir solo se especifica en Sonora para el tema de velocidad excesiva. Para alcohol y conducción, Chiapas, Coahuila, Jalisco, Querétaro, Tamaulipas y Veracruz establecen esta medida. En Michoacán, causar un evento de tránsito por alcohol y conducción es motivo de cancelación de la licencia.

Algunas particularidades muestran que, en Tabasco, las multas por velocidad excesiva, alcohol y conducción, cinturón de seguridad, SRI y uso de casco en motociclistas son conmutables por arresto administrativo. Por último, el sistema de puntos se emplea de manera sistemática en la Ciudad de México para todos los factores analizados.

**CUADRO 2:** Análisis de los distintos criterios utilizados para valorar como adecuada una legislación, según factor de riesgo o protector de lesiones causadas por el tránsito, México 2016

Exceso de velocidad						Alcohol y conducción						Uso de celular al conducir		Cinturón de seguridad			Sístemas de retención infantil (SRI)				Uso de casco en motociclistas			
Entidad federativa	Límite en vía rápida	Límite vía primaria	Límite vía secundaria	Límite zona escolar	Detección	Sanción	Definición	Tasa	Tasa jóvenes	Forma de captación	Mecanismo de control	Sanción	Incluye literalmente el texteo	Sanción	Quién lo usa	Uso	Sanción	Criterio	Tipo de SRI	Certificación	Sanción	Uso	Tipo	Quién debe usarlo	Sanción
**Nivel FEDERAL**	Sí	Sí	NE	NE	No	Sí	Sí	No	No	Sí	Sí	Sí	NA	NA	Sí	No	Sí	NA	NA	NA	NA	No	No	Sí	Sí
**Aguascalientes**	Sí	Sí	No	No	No	No	Sí	No	No	Sí	Sí	Sí	No	Sí	Sí	No	No	No	Sí	No	No	No	No	Sí	No
**Baja California**	NA	NA	NA	NA	NA	NA	NA	NA	NA	NA	NA	NA	NA	NA	NA	NA	NA	NA	NA	NA	NA	NA	NA	NA	NA
**Baja California Sur**	NE	Sí	Sí	Sí	No	No	NA	NA	NA	NA	NA	NA	No	No	No	No	No	NA	NA	NA	NA	No	No	Sí	No
**Campeche**	NE	Sí	Sí	Sí	No	No	No	No	No	No	No	No	No	No	Sí	No	No	No	No	No	No	No	No	Sí	No
**Chiapas**	Sí	Sí	NE	Sí	Sí	Sí	No	No	No	Sí	Sí	No	No	Sí	No	No	Sí	NA	NA	NA	NA	No	No	Sí	Sí
**Chihuahua**	Sí	No	NE	No	Sí	No	Sí	No	Sí	No	Sí	Sí	No	Sí	Sí	No	Sí	No	No	No	Sí	No	No	Sí	Sí
**Coahuila**	Sí	Sí	NE	No	No	Sí	No	No	No	Sí	No	No	NA	NA	No	No	Sí	No	No	No	Sí	No	No	Sí	Sí
**Colima**	Sí	Sí	NE	NE	No	Sí	NA	NA	NA	NA	NA	NA	NA	NA	Sí	No	No	NA	NA	NA	NA	No	No	No	No
**Ciudad de México**	Sí	Sí	No	No	Sí	Sí	Sí	No	No	Sí	Sí	No	No	Sí	Sí	No	Sí	Sí	Sí	No	No	Sí	No	Sí	Sí
**Durango**	NA	NA	NA	NA	NA	NA	NA	NA	NA	NA	NA	NA	NA	NA	NA	NA	NA	NA	NA	NA	NA	NA	NA	NA	NA
**Estado de México**	NE	Sí	NE	Sí	No	Sí	Sí	No	Sí	Sí	Sí	No	NA	NA	No	No	Sí	NA	NA	NA	NA	No	No	Sí	Sí
**Guanajuato**	Sí	Sí	NE	Sí	No	Sí	No	No	No	No	No	No	NA	NA	Sí	No	No	NA	NA	NA	NA	No	No	Sí	Sí
**Guerrero**	NE	Sí	NE	Sí	No	Sí	No	No	No	No	No	No	NA	NA	No	No	Sí	NA	NA	NA	NA	No	No	Sí	Sí
**Hidalgo**	Sí	Sí	NE	Sí	No	Sí	NA	NA	NA	NA	NA	NA	NA	NA	NA	NA	NA	NA	NA	NA	NA	NA	NA	NA	NA
**Jalisco**	NE	Sí	NE	No	Sí	Sí	Sí	Sí	No	Sí	Sí	Sí	No	No	Sí	Sí	Sí	Sí	Sí	Sí	Sí	Sí	Sí	Sí	Sí
**Michoacán**	NE	Sí	Sí	Sí	No	Sí	Sí	No	No	Sí	No	No	No	Sí	No	No	Sí	NA	NA	NA	NA	No	No	Sí	Sí
**Morelos**	Sí	Sí	No	Sí	No	Sí	Sí	Sí	No	Sí	Sí	Sí	NA	NA	Sí	No	No	NA	NA	NA	NA	No	No	Sí	No
**Nayarit**	NE	Sí	Sí	Sí	No	Sí	Sí	No	No	Sí	No	No	NA	NA	NA	NA	NA	NA	NA	NA	NA	No	No	No	Sí
**Nuevo León**	NA	NA	NA	NA	NA	NA	Sí	No	No	No	No	No	NA	NA	NA	NA	NA	NA	NA	NA	NA	NA	NA	NA	NA
**Oaxaca**	NE	Sí	NE	NE	No	Sí	No	No	No	No	No	No	NA	NA	NA	NA	NA	NA	NA	NA	NA	NA	NA	NA	NA
**Puebla**	NE	No	No	Sí	No	Sí	No	No	No	Sí	Sí	No	No	Sí	Sí	No	Sí	No	No	No	Sí	No	No	Sí	Sí
**Querétaro**	NE	Sí	No	NE	No	Sí	No	No	No	No	No	No	No	Sí	Sí	No	Sí	NA	NA	NA	NA	No	No	Sí	Sí
**Quintana Roo**	NA	NA	NA	NA	NA	NA	No	No	No	No	No	No	No	No	Sí	No	No	No	No	No	No	No	No	Sí	No
**San Luis Potosí**	NA	NA	NA	NA	NA	NA	Sí	No	No	No	Sí	No	NA	NA	NA	NA	NA	No	No	No	No	NA	NA	NA	NA
**Sínaloa**	Sí	Sí	No	Sí	No	No	No	No	No	No	No	No	NA	NA	Sí	Sí	No	Sí	Sí	No	No	No	No	Sí	No
**SoNora**	Sí	No	No	Sí	No	Sí	Sí	Sí	No	Sí	Sí	No	No	No	Sí	No	Sí	No	No	No	Sí	No	No	Sí	No
**Tabasco**	Sí	Sí	Sí	Sí	No	Sí	Sí	No	No	Sí	Sí	No	NA	NA	Sí	No	Sí	No	No	No	Sí	No	Sí	Sí	Sí
**Tamaulipas**	NE	Sí	NE	Sí	No	Sí	Sí	No	No	No	No	No	No	Sí	No	No	Sí	No	No	No	No	No	No	Sí	Sí
**Tlaxcala**	NE	Sí	No	Sí	No	Sí	No	No	No	Sí	Sí	No	NA	NA	Sí	No	Sí	NA	NA	NA	NA	No	No	No	Sí
**Veracruz**	Sí	No	No	Sí	Sí	Sí	Sí	No	No	Sí	Sí	Sí	No	Sí	Sí	No	Sí	Sí	Sí	No	Sí	Sí	Sí	No	Sí
**Yucatán**	Sí	No	No	Sí	Sí	Sí	Sí	No	Sí	Sí	Sí	Sí	No	Sí	Sí	No	Sí	No	Sí	No	Sí	No	No	Sí	Sí
**Zacatecas**	Sí	Sí	Sí	Sí	Sí	Sí	Sí	No	Sí	Sí	Sí	No	NA	NA	Sí	No	No	Sí	No	No	No	Sí	No	Sí	No

NE, no especificado; NA, no aplica por no tener disposición específica para el factor de riesgo específico.

*Nota*: cuadro de elaboración propia con base en los resultados presentados.

***Nota:*** figura de elaboración propia a partir de los resultados presentados.

## DISCUSIÓN

Hasta donde sabemos, este es el primer análisis que se realiza a nivel subnacional de la legislación en materia de seguridad vial en México. Los resultados parecen ser desalentadores: ninguna entidad federativa tiene normativas adecuadas para los seis factores analizados. Pese a ello, esta es información de gran utilidad para el país, pues orienta a los tomadores de decisiones nacionales y locales sobre el estado actual y las brechas que hay que subsanar mediante las adecuaciones pertinentes. Aunque algunos criterios utilizados pudieran parecer excesivos, son elementos que ya han sido documentados como esenciales para que la aplicación de la legislación sea efectiva y se traduzca en vidas salvadas, o bien, lesiones y discapacidades evitadas o minimizadas (3, 4). Es importante tener en cuenta que, si bien las legislaciones son un fin en sí mismas porque establecen parámetros que permite contar con reglas claras de tránsito, son además un medio para facilitar el control policial cuando estas no se respetan. Una legislación inadecuada impide un control policial efectivo y desincentiva su cumplimiento. De allí la importancia de concientizar y sensibilizar a las autoridades legislativas sobre la necesidad de ir cerrando brechas entre lo recomendado por la evidencia científica y lo observado en este análisis.

Los resultados presentados brindan además elementos clave para enriquecer la discusión nacional sobre la relevancia de contar con una ley general de seguridad vial que establezca estándares claros y adecuados que puedan ser adoptados por las entidades federativas y sus municipios o una ley federal de seguridad vial que sea de aplicación general. Mientras esto ocurre, es importante tener presente que si en la legislación de las 32 entidades federativas y de las carreteras federales se cubrieran estos factores en forma adecuada y se aplicaran con rigurosidad, esta normativa no sería necesaria. Es por ello que es deber de las autoridades locales y nacionales mejorar las normas de seguridad vial respectivas y así avanzar en la agenda nacional e internacional sobre seguridad vial. Mediante el reconocimiento de esta necesidad, la Secretaría de Salud, a través del “Programa de Acción Específico: Seguridad Vial 2013–2018”, planteó como objetivo proponer un marco jurídico en seguridad vial que incluya los principales factores de riesgo con tres líneas de acción que buscan contribuir al mejoramiento de la normatividad en los tres niveles de gobierno (13).

Aunque perfectible, fue evidente que Jalisco cuenta con la mejor legislación estatal en seguridad vial. Esto es resultado de la disponibilidad política de las autoridades del poder ejecutivo y legislativo de la entidad que catalizaron el trabajo multidisciplinario e intersectorial realizado en el marco de la Estrategia Nacional de Seguridad Vial 2011-2020 y del Programa Global de Seguridad Vial de la Filantropía de Bloomberg. Esta última apoyó con financiamiento los esfuerzos nacionales y estatales, facilitando el acompañamiento cercano de OPS/OMS así como de otros actores internacionales y nacionales (14, 15). Estos avances fueron destacados en el reciente Informe Global (6) que mostró a esta entidad federativa como “caso exitoso”, con resultados promisorios que deberán ser evaluados en el mediano y largo plazos (16–18).

El presente documento tiene algunas limitaciones. No se consideraron todos los elementos importantes para valorar la adecuación de las legislaciones de tránsito descritos por la OMS (12). Tampoco se incluyeron otros factores tales como: licenciamiento para los conductores de vehículos de motor y el uso de casco en ciclistas, entre otros. Es importante enfatizar que para que una legislación sea integral no solo debe ser adecuada, sino aplicarse de manera rigurosa, tema no abordado en este trabajo debido a la escasez de información colectada de forma sistemática y comparable en territorio nacional.

Es importante considerar que este análisis falla en documentar la reglamentación de tránsito en los 2 454 municipios del país. En entidades en donde no existen legislaciones estatales, sería importante valorar si en sus municipios existen y son adecuadas las normatividades en seguridad vial. Se podría dar el caso de municipios con excelente normatividad en seguridad vial en entidades federativas con disposiciones estatales inadecuadas, o viceversa. Se desconoce al momento el número de municipios que no cuentan con reglamentos de tránsito o el porcentaje que se adhieren a las disposiciones estatales y federales de los que sí cuentan con uno. Las entidades federativas interesadas podrían a su vez realizar ejercicios similares en sus municipios prioritarios para hacer un diagnóstico específico que identifique oportunidades de mejora.

Tampoco se abordó aquí hasta qué punto las sanciones establecidas en el país logran realmente su propósito. Se pudo observar que la sanción económica fue la más utilizada con mayor frecuencia, quizá porque es la forma en que los estados y sus municipios recaudan recursos para apoyar la ejecución de otros programas o acciones de gobierno. Estudios futuros podrían analizar en qué medida las sanciones vigentes en las distintas entidades federativas desincentivan las conductas de riesgo. Sería ideal contar con información del tipo o nivel de sanción a partir del cual se logra eficazmente desincentivar comportamientos que atentan la seguridad vial.

Por último, el método empleado podría incorporar algunos sesgos; aunque se trató de minimizar la variación interobservador con la actuación de un solo profesional y se revisaron algunos temas para evaluar la calidad de la información obtenida, no se tiene información precisa de la posible variación intraobservador.

### Conclusiones

El presente análisis contribuye al acervo nacional e internacional al documentar el caso de México en materia de legislación en seguridad vial a nivel subnacional; un insumo más para avanzar en la ambiciosa meta que nos hemos propuesto como país en la Estrategia Nacional de Seguridad Vial 2011–2020 (19).

En general, los factores protectores y los de riesgo se hallan dentro en la normatividad de seguridad vial nacional y estatal. El alcohol y conducción y la velocidad fueron los factores de riesgo con mayor regulación, y el uso de celular es el menos establecido en las disposiciones legales analizadas. Solo en los casos de velocidad en Zacatecas y uso de cinturón de seguridad, SRI y casco en Jalisco, pueden ser consideradas adecuadas. El resto de la normativa federal y de las entidades federativas no se adhiere a las recomendaciones internacionales.

Se observó que las sanciones pecuniarias son las más recurrentes en la normativa, con montos establecidos de acuerdo a la gravedad de la falta y al SMV nacional. El arresto administrativo parece ser de las sanciones menos recurrentes en el tema de la seguridad vial, ya que solo doce entidades lo contemplan en el tema de alcohol y conducción.

### Recomendaciones

Con base en los datos recogidos en el presente estudio, se elaboran las siguientes recomendaciones:

- Establecer en las leyes y reglamentos de tránsito municipales, estatales y federales el nivel máximo de alcohol permitido al conducir en 0,25 mg/L de aire espirado y 0,1 mg/L de aire espirado para conductores jóvenes o noveles.
- Adecuar los límites máximos de velocidad a las recomendaciones de la OMS.
- Incluir la prohibición explícita de hablar o textear al conducir en los distintos ordenamientos jurídicos.
- Conducir análisis al interior de los estados para evaluar en qué medida sus municipios siguen las disposiciones estatales.
- Evaluar en qué medida las sanciones establecidas actualmente desincentivan conductas de riesgo de forma exitosa y, de ser necesario, ajustarlas.

## Agradecimiento.

Los autores desean agradecer el importante apoyo recibido por Alejandro Bernabé López Ávila en la revisión de la legislación y la conformación de la base de datos analizada.

## Declaración.

Las opiniones expresadas en este documento representan el punto de vista de los autores y no necesariamente de la institución en la que laboran ni la política de la *RPSP/PAJPH* y/o de la Organización Panamericana de la Salud.

## Footnotes

Traducción literal de *texting* en inglés. En español la palabra texteo no existe, para efectos de este texto se entiende como la acción de revisar, leer, analizar, escribir o enviar un mensaje de texto a través de un teléfono móvil.

Según el artículo 90 de la Ley Federal del Trabajo, el salario mínimo se define como “la cantidad menor que debe recibir en efectivo el trabajador por los servicios prestados en una jornada de trabajo. Este deberá ser suficiente para satisfacer las necesidades normales de un jefe de familia en el orden material, social y cultural, y para proveer a la educación obligatoria de los hijos” (20).

La moneda corresponde a pesos mexicanos.

Se toma como referencia la tasa de cambio al día primero de enero de 2016, 1 peso equivale a 0,05764 dólares estadounidenses (USD).
